# The economic costs of malaria in four Kenyan districts: do household costs differ by disease endemicity?

**DOI:** 10.1186/1475-2875-9-149

**Published:** 2010-06-02

**Authors:** Jane Chuma, Vincent Okungu, Catherine Molyneux

**Affiliations:** 1 Kenya Medical Research Institute-Wellcome Trust Research Programme, P.O Box, 230, Kilifi, Kenya; 2 Centre for Tropical Medicine, Nuffield Department of Clinical Medicine, University of Oxford, UK

## Abstract

**Background:**

Malaria inflicts significant costs on households and on the economy of malaria endemic countries. There is also evidence that the economic burden is higher among the poorest in a population, and that cost burdens differ significantly between wet and dry seasons. What is not clear is whether, and how, the economic burden of malaria differs by disease endemicity. The need to account for geographical and epidemiological differences in the estimation of the social and economic burden of malaria is well recognized, but there is limited data, if any, to support this argument. This study sought to contribute towards filling this gap by comparing malaria cost burdens in four Kenyan districts of different endemicity.

**Methods:**

A cross-sectional household survey was conducted during the peak malaria transmission season in the poorest areas in four Kenyan districts with differing malaria transmission patterns (n = 179 households in Bondo; 205 Gucha; 184 Kwale; 141 Makueni).

**Findings:**

There were significant differences in duration of fever, perception of fever severity and cost burdens. Fever episodes among adults and children over five years in Gucha and Makueni districts (highland endemic and low acute transmission districts respectively) lasted significantly longer than episodes reported in Bondo and Kwale districts (high perennial transmission and seasonal, intense transmission, respectively). Perceptions of illness severity also differed between districts: fevers reported among older children and adults in Gucha and Makueni districts were reported as severe compared to those reported in the other districts. Indirect and total costs differed significantly between districts but differences in direct costs were not significant. Total household costs were highest in Makueni (US$ 19.6 per month) and lowest in Bondo (US$ 9.2 per month).

**Conclusions:**

Cost burdens are the product of complex relationships between social, economic and epidemiological factors. The cost data presented in this study reflect transmission patterns in the four districts, suggesting that a relationship between costs burdens and the nature of transmission might exist, and that the same warrants more attention from researchers and policy makers.

## Background

Malaria inflicts significant costs on households and on the economy of endemic countries. Evidence also suggests that the economic burden is higher among the poorest in a population and that cost burdens differ significantly between wet and dry seasons [1-5]. What is not clear is whether, and how, the economic burden of malaria differs by disease endemicity.

Malaria endemicity refers to the amount of malaria in a given region; endemicity determines which groups of the population are at highest risk of malaria morbidity [6,7]. Depending on the intensity of transmission, malaria can be stable or unstable [6]. Under stable transmission, the prevalence of infection is relatively high; endemicity is insensitive to environmental change although seasonal variations in transmission occur. Individuals living in areas of stable malaria acquire immunity with age. Very young children who have not yet acquired clinical immunity, and pregnant women, whose immunity to malaria is temporarily impaired, are at highest risk of developing clinical disease [6]. Settings with unstable malaria record high variations in prevalence, immunity is low and there is a high risk of malaria epidemic. In such settings, the lack of frequent exposure to malaria infection early in life delays the acquisition of immunity, and both children and adults remain at high risk of malaria disease when exposed [6,7]. It is estimated that 60% of the African population live in areas with stable malaria transmission where significant immunity has developed by the age of five; 30 percent live in areas of seasonal transmission where immunity is gained later by the age of 10 years; and 10 percent live in areas of unstable transmission that are prone to epidemics. In 2007, 2.37 billion people globally lived in areas at risk of *Plasmodium falciparum *transmission; 1 billion lived under unstable malaria risk [8,9].

Endemicity plays an important role in malaria morbidity and mortality [7]. Various studies document differences in malaria prevalence by transmission intensity [8,10-14], but only a minority have explored variations in social and economic aspects of the disease. The limited evidence available suggests that a relationship exists between disease endemicity, treatment-seeking patterns and illness perceptions [11,15-17], but whether these variations translate into cost differences remains unclear. Amin *et al *[15] noted significant differences in malaria prevalence, treatment-seeking patterns, and timing of treatment among children aged below five years in Kenyan districts of different transmission intensity. The use of the informal retail sector was significantly lower in the district of intense perennial transmission than in other districts, and significantly more fevers were treated in a highland transmission district than in other districts. Fevers reported in the highland transmission district were more likely to be treated within the first 24 hours, and in the formal sector [15]. The authors attributed differences in fever prevalence to transmission patterns but did not highlight any potential role of transmission in treatment-seeking decisions. Elsewhere, the risk of malaria morbidity among Kenyan school going children was reported to differ by intensity of transmission [11]. Perceptions of malaria also differed; malaria was perceived as a life-threatening illness in a highland transmission district and as a mild everyday illness in a district with intense transmission [16]. While none of these studies explored variations in cost burdens, the findings suggest that transmission patterns can influence perceptions of illness, treatment-seeking decisions and ultimately cost burdens.

The need to take into account epidemiological differences when estimating the social and economic burden of malaria is well recognized. Tanner and Vlassoff note that malaria endemicity can influence health-seeking behavior, illness perceptions, and the socio-economic burden of the disease and highlight the need to consider such differences when designing malaria control interventions [17]. Goodman *et al *[18] call for an urgent need to account for epidemiological differences between and within countries, and recommend that such differences be accounted for when estimating the economic burden of malaria and in policy designs. Other authors recognize the existence of such differences [19-22], but studies on the economic burden of malaria have generated little empirical evidence to validate these theoretical propositions.

### Potential pathways through which transmission patterns impact on cost burdens

**Figure 1:**
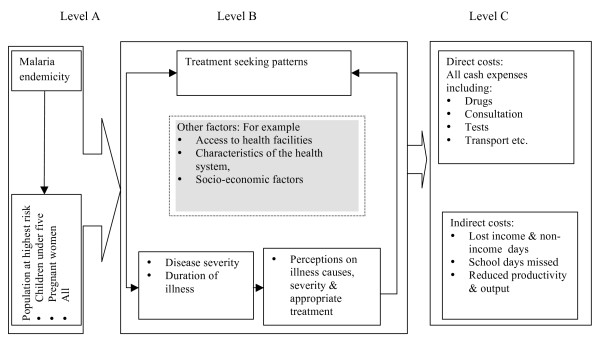
**Malaria transmission and cost burdens: a summary of potential pathways**.

Based on the literature, a number of pathways through which transmission differences can influence cost burdens either directly or indirectly have been identified (Figure 1). Starting from Level A, malaria endemicity determines the population at highest risk of developing clinical disease in a particular setting. People perceive malaria differently depending on the population at highest risk of malaria morbidity and their exposure to clinical disease; these perceptions influence treatment-seeking decisions (Level B). For example, individuals living in areas of unstable transmission may perceive malaria as a deadly disease- and thus are more likely to seek treatment quickly and in the formal sector-compared to those living in stable transmission areas, where malaria is an 'everyday illness' and, therefore, perceived as less severe [11,15,17]. The large shaded box in the centre shows that in addition to endemicity, treatment-seeking behavior is influenced by other factors, for example access to health care facilities, the health system and social networks. These factors are not explored further in this paper, although different aspects of the same have been published elsewhere [23,24].

Households incur direct and indirect costs due to malaria (Level C). Direct costs such as treatment and transport costs are largely influenced by the type of treatment sought, disease severity and duration of illness [1,15]. In generally, treatment for malaria costs more in the formal sector than in the informal sector, although the effectiveness of the latter is questionable. Private facilities are the most expensive sources of malaria treatment [1,15]. Indirect costs due to inability to conduct livelihood activities due to illness can be higher in areas of unstable transmission than elsewhere. For example, the economic burden may be lower in settings where malaria is concentrated among young children than in settings where both adults and children are equally vulnerable to malaria. Malaria affects the quality of labour; it can lead to absenteeism, and even though an acute malaria attack might not prevent people from working, it can reduce the quality of productivity and output [18]. Absenteeism from work can lead to income losses, particularly in the informal sector, where income is largely dependent on the number of productive hours. School going children living in areas of low transmission report more malaria than those in higher transmission settings [11]. Although few studies have documented the impact of malaria on school attendance, it is estimated that students loose between 3-12 days per year due to malaria [25]; malaria accounts for 8 percent of school absenteeism [10]; and the disease can dramatically affect school attendance [26]. Clearly, these variations can translate to significant differences in the economic burden of malaria between settings. This study sought to contribute towards filling this gap in evidence on costs by exploring whether, and how, malaria cost burdens reflect transmission patterns in four Kenyan districts.

### Malaria transmission in Kenya

Kenya has a diverse climate and ecology supporting the entire range of stable and unstable transmission conditions. Malaria is a major public health problem, and in many parts of the country, pregnant women and children below the age of five are most at risk of developing clinical disease [6]. It is estimated that in 1997 eight million Kenyans were exposed to unstable malaria, and five million were exposed to a low risk of stable malaria. Approximately four million people, including 677,000 children, were living in areas of high transmission [6].

Using data collected by Snow *et al *[6], the Division of Malaria Control (DOMC) has categorized Kenyan districts into five categories of disease ecology. These include: (1) lake-side endemic: referring to districts close to Lake Victoria where transmission occurs throughout the year; (2) coastal endemic, which is similar to the lake side endemic, but differs in that transmission and risk of infection exhibit stronger seasonality; (3) highland epidemic-prone districts, where there is limited transmission, but the districts are prone to epidemics due to variations in rainfall and temperatures between years; (4) arid epidemic prone-districts, where only communities living close to water sources are at the risk of developing clinical disease, transmission is extremely low, and residents do not develop immunity; and (5) low-risk districts that experience almost no risk of malaria infection. The variation offers an opportunity to explore the extent to which the economic burden reflects malaria endemicity within a single country. This study explores the extent to which malaria cost burdens reflect disease endemicity in four Kenyan districts. While existing costs data have been extremely useful for informing policy, understanding the role of disease endemicity in the economic burden of malaria is important for defining priorities and for targeting interventions equitably and efficiently.

## Methods

### Study area

**Figure 2:**
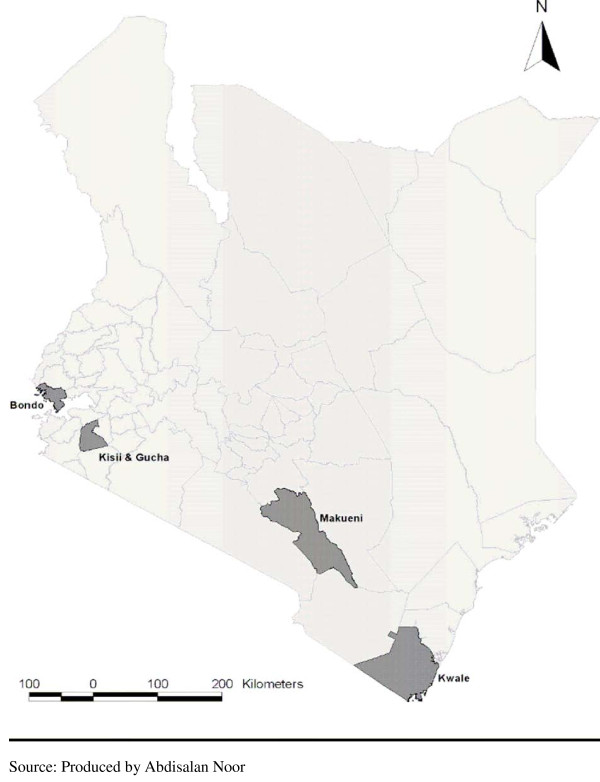
**A map of Kenya showing the location of the study districts**.

The study was conducted in four districts purposively selected to represent the different malaria ecologies in the country. The districts have also been involved in a wide range of studies conducted by KEMRI-Wellcome Trust researchers, in collaboration with the DOMC, to monitor and evaluate targets set as part of the national malaria strategy [27]. Each district represents one of the four major malaria ecologies in Kenya: (1) Gucha district on the western highlands, represents an area with low, unstable transmission that is prone to epidemics; (2) Kwale on the coast of Kenya, a semi-arid district with seasonal, intense transmission representing the coastal endemic regions; (3) Bondo district, on the shores of Lake Victoria, an area of perennial, intense transmission, representing Lake side endemic districts and; (4) Makueni in the Eastern part of Kenya, a semi-arid district with low acute seasonal transmission. Prevalence of *P. falciparum *infection among children aged below 10 years has been recorded as lowest in Makueni and Gucha districts (prevalence of 16% and 17% respectively) and highest in Bondo and Kwale districts (prevalence of 58% and 55% respectively) [15,28]. Malaria accounts for about half of all out-patient visits among children in all districts. A map of Kenya showing the location of the districts is shown in Figure 2.

The districts are also some of the poorest in the country: the percentage of individuals living below the poverty line is estimated as 63% in Kwale; 62% in Makueni; 74% in Gucha and; 70% in Bondo [29]. According to the 1999 population census, Gucha district is the most densely populated of the four districts, followed by Bondo, Makueni and Kwale respectively. Children under five account for less than 20 percent of the population in all districts [30]. Agriculture is the main income generating activity in all districts, although Kwale has a significant tourism sector. Each district is served by one district hospital located at the district headquarters, but all the Enumeration Areas (EAs) where data were collected are located far from the district headquarters. The main health care providers in the study villages are public primary health providers (dispensaries and health centers), except for Bondo, where a faith-based hospital was located in close proximity to the study setting. There were no private hospitals operating in close proximity to the study settings.

### Data collection methods

Data presented in this paper were part of a wider study conducted to explore barriers to effective malaria treatment and prevention in Kenya. A multi-stage sampling approach was adopted to select the survey households: First, locations-the 2^nd ^lowest administrative unit in Kenya-were selected using poverty indicator maps, generated for the entire country by the Kenya National Bureau of Statistics [29]. The maps classified locations within each district into poverty quintiles based on the percentage of the population living below the international poverty line of USD 1 per day. All locations which fell within the two poorest quintiles in each district were identified. Four enumeration areas (EAs) located in the two poorest quintiles in each district were then selected using simple random sampling approach. The homestead list was updated and all homesteads in the EAs were selected to participate in the study. All households in the homestead were included in the study, resulting to a total of 708 households (n = 179 Bondo; 204 Gucha; 184 Kwale and; 141 Makueni). A minimum of 140 households per district was estimated as sufficient to detect differences in the proportion of households incurring cost burdens greater than 10% of household expenditure, a figure taken in the literature to indicate catastrophic spending. A total of 20 households declined to participate in the study.

A homestead was defined as a collection of adjacent or nearby households with a single individual as a head. Homestead members are usually related through kinship ties, and typically comprise of the homestead head, his/her sons and their families. A household was defined as a group of people living in the same homestead and who shared a common source of food and/or income. There could have been more than one household within a homestead because residents did not always share income or ate from the same pot. Additional information on the sampling approach is presented elsewhere [23,24].

Seasonality is a key factor in malaria transmission [6] and it influences illness perceptions [31], and cost burdens [1]. All the districts experience long rains between March to May and short rains occur around November to December. Previous work conducted by the authors in a rural and an urban area of the Kenyan coast revealed that treatment-seeking patterns and cost burdens differed between the wet and dry seasons, with households reporting significantly higher direct and indirect costs in the wet season compared to the dry season [1,32]. To address possible seasonality differences between districts, all data in the four districts were collected at the same time during the peak malaria transmission season when people were more likely to report fever. Reported fevers and costs burdens in the same districts may be significantly lower in the dry season when malaria transmission is lowest.

Using a recall period of two weeks, the cross-sectional household survey collected data on socio-demographic indicators, treatment-seeking patterns, direct and indirect costs of malaria. The study used self-reported fever as the main indicator of malaria, although not all fevers are malaria [8]. A semi-structured questionnaire was administered in the local language to the household heads or spouses or, in their absence, another senior member of the household. The history of fevers in the last two weeks was taken for all household members who reported illness in the two weeks preceding the survey. All respondents were aged above eighteen years.

Direct costs were defined as all cash spending arising due to a fever episode including, spending on drugs or herbs, consultation fees, diagnostic tests, transport and prescription books. Indirect costs were defined as inability to conduct income and non-income generating activities for the ill person and the caretaker. For indirect costs, ill individuals were asked to report the number of days they were unable to conduct income and non-income activities due to illness, and whether a caretaker assisted them during the illness periods. In cases where the ill individual was assisted by a caretaker, questions were asked regarding the ability of the care taker to conduct income and non-income generating activities during the caring period. School going children were asked to report the number of school days missed due to reported fever. Other information gathered included treatment-seeking patterns, health care charges and barriers to access.

Expenditure data were collected for various items found relevant for the study settings. They included spending on food, cooking fuel, cleaning, lighting, rent, transport, health and health care, remittance, education, debt repayment, contribution to community groups and churches. An additional question to capture any other spending not covered in these categories was included. Different recall periods were applied for different items to minimize recall bias. For items that were likely to be purchased on a daily basis (for example, food, soap), households were asked how much they spent during the last week and whether or not that time period represented a 'normal' week in terms of spending pattern. A monthly recall period was applied for less frequent spending items like education, debt repayment and rent.

### Data analysis

Quantitative data were double entered into Microsoft visual FoxPro (version 9.0) and transferred to STATA (version10) for analysis. To account for unequal probabilities of EAs selection, data were adjusted for clustering in STATA, with the EA taken as the primary sampling unit.

Days lost due to reported fever were valued into monetary terms using an average daily income rate, estimated from consumption expenditure data, which were collected as part of the cross-sectional survey. This rate was estimated as KES 60 (USD 0.8) per adult per day. Experiences of using this approach shows that the estimated daily expenditure rates compare well to the wage rates in the informal labor market [1,32,33]. A similar rate was applied to value days lost in the four districts to enable meaningful comparison. Non-income days lost were not valued into monetary terms because of the potential to overestimate costs arising from 'mild' illnesses. Total costs per household were estimated by summing the direct and indirect costs.

Costs are expressed per household per month because households are the locus of much decision-making, many resources are allocated within the household and decisions to seek treatment and sources of finance involve discussions between household members [34]. Moreover, direct and indirect illness costs are borne largely by healthy members of households rather than by the sick individual [35]. Costs were converted into monthly costs by first dividing the costs reported in the survey by two to arrive at a weekly estimate; the weekly estimates was multiplied by 52 to arrive at an annual estimate; and the annual estimate was divided by 12 to arrive at a monthly estimate.

As expected, cost data were highly skewed to the right. These data were transformed into logarithm in order to fulfill the assumption of normality usually required for the use of parametric tests such as ANOVA and t-test. Logarithmic transformation has been widely applied in the cost of illness literature because the geometric mean is always lower than the arithmetic mean, therefore addressing the problem of a possible overestimation of the mean when data are not transformed [36-40]. The chi-square test was used to compare differences in proportions. A limitation of ANOVA is that it only reports differences in at least one mean but it does not provide information on where the significant differences exist. Bonferroni and Scheffe multiple comparison tests were conducted to examine differences between means for each pair of districts.

In addition to comparing differences across the four districts, the districts were collapsed into two categories reflecting *P. falciparum *infection prevalence among children under five based on epidemiological studies conducted previously in the study districts. Makueni and Gucha were classified as districts of 'low' transmission because they have the lowest plasmodium falciparum infection prevalence among children, while Bondo and Kwale districts were categorized as 'high' transmission districts because they reported the highest plasmodium falciparum infection prevalence [15,28].

### Ethical issues

Ethical approval was obtained from the Kenya Medical Research Institute (SSC no. 964) and from the World Health Organization Ethics Review Committee (ID A50045). Informed consent was sought from the household head, from all adults participating in the survey, and from guardians in cases where illnesses were reported among children.

## Results

### Socio-demographic characteristics and self reported fever among survey households

Table 1 provides a summary of the socio-demographic and economic characteristics of survey households relevant to this paper. The findings show no significant differences in the composition of the survey households for age and gender, but significant differences exist in terms of main income generating activities (p < 0.005). Mean monthly expenditure in all districts was below the international accepted poverty line of USD 1.25 per day.

**Table 1:** Socio-demographic characteristics of survey households

Variable	Bondo n = 901 (%)	Gucha n = 1048 (%)	Kwale n = 1329 (%)	Makueni n = 936 (%)
Age				
• < 5 years	141 (15.7)	198 (18.9)	252 (19.0)	138 (14.7)
• > = 5 years	760 (84.3)	850 (81.1)	1077 (81.0)	798 (85.3)

Gender				
• Male	467 (51.8)	569 (54.3)	711 (53.5)	447 (47.8)
• Female	432 (41.2)	479 (45.7)	618 (46.5)	489 (52.2)

Occupation for adults				
• Agriculture	260 (62.8)	353 (77.1)	392 (76.4)	281 (66.9)
• Non-agriculture	154 (37.2)	105 (22.9)	121 (23.4)	169 (33.1)

The proportion of individuals reporting fever in the two weeks prior to the survey ranged from 18.4% in Makueni to 32.1% in Kwale (Table 2). There were significant differences in the proportion of children under five reporting fever between districts (p < 0.005): the highest proportion of children aged below five years reporting fever was recorded in Bondo (44.7%), and the lowest in Gucha and Makueni districts. The duration of an illness episode among individuals who reported full recovery on the date of interview differed significantly between districts (p < 0.005): the largest mean episode duration of six days was reported in Gucha and Makueni districts, and the lowest mean duration was reported in Bondo. In all districts, most fever episodes among children aged below five years were perceived as severe, ranging from 81% in Bondo to 65.2% in Kwale (p = 0.025), but more fever episodes among older children and adults were perceived as severe in Gucha and Makueni districts compared to Bondo and Kwale districts (p < 0.001).

**Table 2:** Distribution of self-reported fever across districts

Variable	Bondo n = 901 (%)	Gucha n = 1048 (%)	Kwale n = 1329 (%)	Makueni n = 936 (%)
Proportion of self-reported fevers				
• < 5 years	63 (44.7)	43 (21.7)	105 (41.7)	30 (21.7)
• > = 5 years	199 (26.2)	170 (20.0)	322 (29.9)	142 (17.8)
• All	262 (29.1)	213 (20.3)	427 (32.1)	172 (18.4)

Self-reported fever by gender (age > = 15 years)				
• Male	46 (25.3)	56 (21.3)	102 (33.4)	39 (21.3)
• Female	30 (17.8)	31 (15.1)	59 (29.2)	28 (15.7)

Fever perceived as severe*				
• < 5 years	51 (81.0)	31 (72.0)	73 (65.2)	20 (66.7)
• > = 5 years	97 (48.2)	129 (75.6)	184 (56.4)	86 (60.4)

Proportion of fevers treated				
• < 5 years	52 (89.7)	44 (93.6)	102 (81.1)	27 (90.0)
• > = 5 years	190 (94.5)	150 (89.3)	299 (91.2)	116 (82.9)

Proportion of fevers treated in the formal sector				
• < 5 years	25 (43.1)	22 (46.4)	38 (34.0)	20 (66.7)
• > = 5 years	62 (30.9)	40 (23.8)	78 (23.8)	63 (45.0)

The proportion of fevers where an action was taken in response to an illness was relatively high in all districts (ranging from 84.6% in Kwale to 93.7% in Bondo). There were no significant differences in the proportion of fevers treated among children under five and older groups in any of the districts. The informal sector was the main source of treatment for fevers in all districts, although significantly more fevers in Makueni were treated in the formal sector compared to other districts (p < 0.005).

### Direct and indirect costs of self-reported fever

A total of 778 fevers were treated using drugs bought from the shops (n = 160 Bondo; 159 Gucha; 258 Kwale; 89 Makueni). The highest mean cost for drugs bought from the shops per action was reported in Gucha (KES 60), and the lowest in Kwale (KES 32). This difference was statistically significant (ANOVA, p < 0.0001). Median drug costs per action were significant between districts (Kruskall-wallis, p < 0.0001), with the highest median drug costs per action reported in Gucha district (KES 50), and lowest in Kwale (KEs 20). Additional analysis revealed that significant differences in mean costs of shop drugs per action existed between Gucha and Bondo districts, and between Gucha and Kwale. Mean facility-based costs, i.e. amount of money paid at the health facility per action taken at public dispensaries, were highest in Bondo and Gucha (KES 117 and 116 respectively), and lowest in Makueni and Kwale (KES 75 and KES 70 respectively), but this difference was not statistically significant.

The majority of adults reporting fever were economically active (89.0%); 83.3% of the economically active ill individuals reported they could not conduct their income generating activities normally due to the illness (ranging from 76.7% in Makueni to 90.7% in Gucha). Table 3 shows the mean number of days affected by a fever episode. Among adults reporting fever, the mean number of income generating days affected per fever episode differed significantly between districts (p < 0.001) with the lowest mean number of income generating days lost reported in Kwale and the highest in Gucha. School going children in Gucha reported missing the highest number of school days due to reported fever (p = 0.022).

**Table 3:** Mean number of days affected by fever for ill individuals and caretakers

	Bondo	Gucha	Kwale	Makueni	P value (ANOVA)
Income days affected for ill adults	5.1	7.8	3.9	7.2	0.000

Income days affected for caretakers	3.9	5.9	3.5	5.4	0.000

School days affected	3.4	4.8	3.6	4.0	0.0222

The distribution of direct and indirect costs expressed as monthly costs per household reporting at least one fever episode showed there were no significant differences in the mean direct costs per household between districts (p = 0.1988). Direct costs were lower than indirect costs in all districts (Table 4); this difference was highest in Gucha where indirect costs were more than three-times that of direct costs. Indirect costs were significantly different between the four districts (p < 0.001). Further tests revealed that the highest differences were observed between Gucha and Bondo (p < 0.001), and Makueni and Bondo (p < 0.001). Significant differences were also observed between Kwale and Gucha; and Makueni and Kwale (p < 0.005). Indirect costs did not differ significantly between Makueni and Gucha (p = 0.981). Combining both direct and indirect costs, mean total costs were significantly higher between districts with the highest costs reported in Makueni (KES 1449) and lowest in Bondo (KES 684). When costs are expressed as a proportion of household expenditure, the results reveal a similar pattern between districts. Mean direct costs as a percentage of monthly expenditure ranged from 5.1% in Kwale to 9.9% in Makueni. Indirect costs ranged from 6.8% in Kwale to 19.8% in Gucha. Households in Gucha and Makueni reported the highest total mean costs burdens of 27.3% and 28% respectively.

**Table 4:** Mean monthly cost per household for households reporting fever

Variable	Bondo n = 146	Gucha n = 123	Kwale n = 149	Makueni n = 85	P value (ANOVA)
Direct costs in KES	310 (4.2)	296 (4.0)	290 (3.9)	464 (6.3)	0.1988
(USD) [Range]	[0-4620]	[0-3420]	[0-3620]	[0-5240]	

Indirect costs in KES	374 (5.1)	893 (12.1)	499 (6.7)	985 (13.3)	0.0000
(USD) [Range]	[0-2040]	[0-5520]	[0-5640]	[0-6000]	

Total costs in KES	684 (9.2)	1189 (16.1)	789 (10.7)	1449 (19.6)	0.0000
(USD) [Range]	[0-4980]	[0-6820]	[0-7920]	[0-10080]	

Districts were collapsed into two categories representing 'high' and 'low' transmission based on *P. falciparum *infection data from the districts [28]. The results presented in Table 5 show that Gucha and Makueni, the districts representing 'low' transmission, reported significantly higher mean indirect and total costs of KES 932 and 1296, compared the districts representing 'high' transmission, which had mean indirect and total costs of KES 438 and 738 respectively (p < 0.001). Differences between mean direct costs were not significant (p = 0.271).

**Table 5:** Mean monthly costs per household by transmission pattern

	'Low' transmission (Gucha and Makueni districts: n = 208)	'High' transmission (Bondo and Kwale districts: n = 295)	P value (ttest)
Direct costs in KES (USD)	364 (4.9)	299 (4.0)	0.271
[95%, Confidence Interval]	[276.0-452.8]	[225.3-374.0]	

Indirect costs in KES (USD)	932 (12.6)	438 (5.9)	0.000
[95%, Confidence Interval]	[766.3-1096.0]	[364.4-511.0)	

Total costs in KES (USD)	1296 (17.5)	738 (10.0)	0.000
[95%, Confidence Interval]	[1089.7-1509.4]	[661.6-858.2]	

## Discussion

This paper set out to explore whether malaria costs burdens differ by transmission patterns. Potential pathways through which malaria transmission patterns impact on cost burdens were identified. Here the discussion demonstrates the extent to which the findings support the notion that endemicity is an important determinant of malaria cost burdens.

There were differences in self-reported fevers between the four districts. Self reported fevers among children below five years of age were highest in Bondo and Kwale, and lowest in Makueni and Gucha. These findings reflect the different transmission patterns represented by the four districts, with Bondo, a district of intense perennial transmission reporting the highest proportion of fevers among children under five, while Gucha and Makueni, the districts of low transmission reporting the lowest proportion of fevers among under fives. Although this study does not estimate prevalence of *plasmodium falciparum *infection, the findings portray a similar pattern with those reported in large scale epidemiological studies conducted in the same districts [6,15,28].

Fever episodes among adults and children over five years of age in Gucha and Makueni districts lasted significantly longer than episodes reported in Bondo and Kwale districts. A wide variation in number of days a fever episode lasts has been reported, ranging from 1 day in Nigeria to 18 days in Ethiopia [2,19,41]. However, the extent to which these differences relate to the nature of disease endemicity is not clear. Perceptions of illness severity also differed between districts: more fevers reported among older children and adults in Gucha and Makueni districts (highland transmission and low acute seasonal transmission respectively) were reported as severe, compared to other districts. Such differences in perceptions might relate to the frequency of malaria morbidity and the consequent development of immunity, as has been reported in other studies in Kenya and elsewhere [11,17]. The implications of differences in perceived severity of fever by endemicity for cost burdens have not been previously documented.

Direct and indirect costs in all districts were high and worrying considering the poverty levels in these settings [30]. Indirect costs differed significantly between districts and were higher than direct costs in all districts. Districts that reported the lowest direct costs did not necessarily record the lowest indirect costs and vice versa. Higher indirect costs have been reported in other settings [1,4,42,43], and this pattern reflects the potential impact of malaria on households income and their future economic development [1]. Indirect costs were highest in Makueni and Gucha (the low transmission districts) and lowest in Kwale (the seasonal intense transmission district). While many factors may influence levels of indirect costs, these data support the hypothesis that high indirect costs in Gucha and Makueni, at least in part, are linked to disease endemicity. Adults and children in these districts are at high risk of developing clinical disease; most fever episodes were reported as severe and adults took more time off their income generating activities, potentially leading to higher income losses, than in the other districts. In the 'high' transmission districts (Bondo and Kwale) illnesses among adults were reported as less severe and although caretakers took time off from work to take care of ill children, their income generating activities were not affected by the illness.

Some studies have reported that school-going children living in low transmission areas miss significantly more days per fever episode compared to those living in higher transmission areas [11]. There were significant differences between districts in the number of school-going days missed by children reporting fever in this study. It is however likely that children living in the highland district of Gucha would have reported missing more school days had the study been conducted during an epidemic.

The potential role of disease endemicity in cost burdens has been documented by various authors [18,44,45]. It is argued that in highly endemic regions, where most illnesses are among infants and children, the impact of malaria on labour productivity and income losses might be lower in low transmission settings. The potential effects of malaria on cognitive skills may have long term impacts on human capital and future earning capacity [46-48], although this impact has not been estimated.

The need to estimate the economic burden of malaria cannot be overemphasized. Cost data have been extremely useful in informing policy. For example, cost data have been widely used to estimate the potential benefits of malaria control and to inform decisions on cost-effectiveness of malaria control compared to other health care interventions. Although widely applied, these data are limited in their generalizability because they relate to specific transmission settings [18,19]. Moreover, macro-economic estimates of the economic burden of malaria are based on combining results from studies conducted in different settings, which hardly account for transmission differences. The value and generalizability of cost data could be strengthened by taking into account geographical and epidemiological variations between and within countries. Such data can inform the modelling of cost-effectiveness of interventions across different epidemiological settings, and inform the allocation of scarce resources to settings that are at high risk of adverse economic burden of the disease.

## Limitations

There are several limitations to this study. First, the study was conducted in the poorest regions of the districts and the findings might not be generalizable to other less poor settings with similar endemicity in Kenya. Second, the study used self-reported fever to indicate malaria. Although not all fevers are malaria, treatment-seeking patterns are highly influenced by perceptions of the disease, and rural households in malaria endemic settings often associate fever with malaria. Moreover, malaria diagnosis in primary health care facilities in Kenya is often based on symptoms rather than parasite tests, implying that individuals (particularly children) presenting with fever at public healthcare facilities in Kenya are likely to be treated for malaria. Third, a single rate was applied to value days lost due to fever episodes based on an estimate of mean daily per capita expenditure. The labor markets in the four districts are likely to differ, although differences in mean monthly expenditure are likely to be insignificant. Fourth, although Gucha district is prone to epidemics, the findings presented in this paper do not represent an epidemic situation and costs burdens could have been higher had the survey been conducted during an epidemic. Finally, the data presented in this paper do not allow for a conclusion that observed cost patterns are entirely due to transmission differences. Other factors could have contributed to the cost differences observed between districts. For example, the social-cultural context influences the loss of income days for caretakers [1,32,49]: in communities where labor substitution is common and where households live as part of extended families, caring for sick individuals can easily be shared among household members without necessarily affecting their ability to conduct the income generating activities. Indirect costs in such settings may be lower, irrespective of the disease endemicity. Nonetheless, the findings did show that cost might differ by disease endemicity and they highlight the need for more systematic studies that are well designed to control for other confounding factors. Future research on this topic should apply a framework such as the one presented in this paper to provide a fuller picture of the role of endemicity on the economic burden of malaria, and conduct path analysis to determine associations between latent variables.

## Conclusions

The authors are not aware of any study that documents differences in household costs of malaria by nature of endemicity. Cost burdens are the product of complex relationships between social, economic and epidemiological factors. However, the fact that the cost data presented in this study reflect, to some extent, transmission patterns in the four districts suggests that a relationship between costs burdens and the nature of transmission might exist, and that the same warrants more attention from researchers and policy makers.

## Competing interests

The authors declare that they have no competing interests.

## Authors' contributions

JC and CM were involved in the conception and design of the study. JC and VO were involved in the data collection, analysis, and writing up. JC wrote the first draft; all authors commented on drafts, read and approved the final manuscript.
